# A retrospective observational study investigating the factors associated with right heart failure in patients with primary acute pulmonary embolism and deep vein thrombosis

**DOI:** 10.1002/jgf2.305

**Published:** 2020-03-04

**Authors:** Satoshi Kobayashi, Makoto Muto, Hitoshi Yabe, Masashi Imao, Yukinori Okada

**Affiliations:** ^1^ Department of Radiology Saitama Rehabilitation Center Ageo Japan; ^2^ Department of Radiology Saitama Cardiovascular Respiratory Center Kumagaya Japan; ^3^ Graduate School of Health Science Suzuka University of Medical Science Suzuka Japan; ^4^ Division of Cardiology Saitama Cardiovascular Respiratory Center Kumagaya Japan; ^5^ Division of Health Sciences Graduate School of Medical Sciences Kanazawa University Kanazawa Japan; ^6^ School of Radiological Sciences Faculty of Health Science Gunma Paz University Takasaki Japan; ^7^ Department of Radiology St. Marianna University School of Medicine Kawasaki Japan

**Keywords:** CT pulmonary angiography, deep vein thrombosis, indirect CT venography, pulmonary embolism, right heart failure, ultrasonography

## Abstract

**Background:**

The relationship between the risk of right heart failure in primary acute pulmonary embolism after embolization and the residual thrombus sites in the pelvis and lower limbs is not clear.

**Methods:**

This single‐center retrospective observational study examined the results of contrast‐enhanced computed tomography and venous ultrasonography of patients with primary acute PE and DVT. We assessed the association between the occurrence of right heart failure and age; gender; pulmonary thrombosis distribution; most proximal site of deep vein thrombosis in the soleal vein, inferior vena cava (IVC), or common iliac vein (CIV); DVT distribution; and malignancy using univariate and multivariate logistic regression.

**Results:**

In all, 77 of 165 patients were male (mean age: 65.1 ± 13.7 years). Right heart failure occurred in 53 patients (32.1%). Multivariate analysis revealed that the odds ratio (OR) for right heart failure was significantly lower in patients with the most proximal site of DVT in the IVC/CIV (OR = 0.07, 95% confidence interval [CI] 0.01‐0.62, *P* = .017), while it was significantly higher in females (OR = 2.51, 95% CI 1.05‐6.01, *P* = .039), and in patients who exhibited the presence of bilateral venous thrombosis (OR = 3.89, 95% CI 1.60‐9.48, *P* = .003).

**Conclusion:**

A significant factor involved in PE without right heart failure was the most proximal site of DVT in the IVC/CIV, and significant risk factors associated with PE with right heart failure were more prevalent in females and in patients who exhibited the presence of bilateral venous thrombosis.

## Abstract 2

The objective of this study was to examine some factors associated with PE with right heart failure in patients with primary acute PE and DVT after embolization, especially the location of residual thrombus in the lower limbs. Therefore, retrospective examination was performed based on the findings of contrast‐enhanced computed tomography (CT) and venous ultrasonography of the lower limbs. A significant factor involved in PE without right heart failure was the most proximal site of DVT in the IVC/CIV, and significant risk factors associated with PE with right heart failure were female gender and the presence of bilateral venous thrombosis.

## 1 INTRODUCTION

Acute pulmonary embolism (PE) can be caused by the sudden blockage of a pulmonary vessel by a clot that has detached from a vein. Severe PE may result in acute right heart failure, causing the patient to go into shock. In the majority of PE cases, the embolic source is the deep veins of the lower extremities.^1^, ^2^ PE usually forms when part of a thrombus detaches and makes its way through the vein toward the heart.^3^ After an embolus has broken off, the venous location of the thrombus that remains in the leg that is closest to the heart is known as the most proximal site of DVT.^4^ Acute PE is diagnosed and treated based on the severity grading system described in the guidelines for the diagnosis, treatment, and prevention of PE and DVT which comprises hemodynamic findings and signs of right heart overload on echocardiography.^5^ According to this classification system, PE is graded on a four‐point scale as (1) nonmassive, (2) submassive, (3) massive, or (4) cardiac arrest or circulatory collapse. Right heart failure is included in the diagnostic criteria for submassive and massive PE, as well as PE leading to cardiac arrest or circulatory collapse since the prognosis and recurrence rate of these conditions depend on of the presence of right heart overload.^6^, ^7^ Risk factors for acute submassive PE with right heart overload have been reported in previous studies.^8^ In addition, there are several reports retrospectively examining the presence of right heart overload in patients with acute PE after embolization and most proximal sites of residual DVT in the lower limbs.^3^, ^9^ Ohgi et al reported that the commonest most proximal site leading to severe acute PE is the femoral vein.^3^ In contrast, Ro et al's^9^ investigation of the most proximal site of residual thrombus in the lower extremities of 100 patients who died of acute PE found that 63% of sites were near the popliteal vein. However, these studies did not specifically analyze using rigorous statistical analysis. In addition, the inferior vena cava (IVC) was not included in the investigation site of the deep vein in these studies. Therefore, the relationship between the risk of right heart failure in primary acute PE after embolization and the residual thrombus site in the lower limb is not clear. The objective of this study was to examine the factors associated with right heart failure in patients with primary acute PE and DVT after embolization, especially the location of residual thrombus in the lower limbs. Therefore, retrospective examination was performed based on the findings of contrast‐enhanced computed tomography (CT) and venous ultrasonography of the lower limbs. In this study, a significant factor involved in PE without right heart failure was the most proximal site of DVT in the IVC/CIV, and significant risk factors associated with PE with right heart failure were female gender and the presence of bilateral venous thrombosis. If DVT is suspected from the symptoms, venous ultrasonography of the lower limb may be performed before contrast‐enhanced CT. In a situation where the presence of PE and right heart failure has not been confirmed by testing, if venous thrombus on both legs is present on venous ultrasonography of the lower limb, the possibility of right heart failure because of PE complications can be considered. It could be used as a criterion to decide whether or not to perform contrast‐enhanced CT and echocardiography.

## 2 MATERIALS AND METHODS

### 2.1 Participants and exclusion criteria

This was a single‐center retrospective observational study. The study was conducted at Saitama Cardiovascular Respiratory Center, Saitama, Japan, a 319‐bed tertiary emergency medical institution. The study was approved by the Ethics Committee of our institution (approval number 2 014 010); the need for informed consent was waived because of the retrospective nature of the study.

All PE or DVT patients diagnosed at our hospital from March 2006 to May 2016 were eligible for inclusion in this study. The patients included outpatients and hospitalized patients (in our hospital or other hospitals). All patients underwent CT pulmonary angiography (CTPA) and indirect CT venography using a multidetector scanner,^10^ in addition to venous ultrasonography of the lower limbs and echocardiography. All tests were conducted at roughly the same time (mean time between tests: 1.3 days).

If PE was suspected from the symptoms, contrast‐enhanced CT was performed before venous ultrasonography of the lower limbs. Immediately after PE was detected by contrast‐enhanced CT, venous ultrasonography of the lower limbs confirmed the presence of DVT. On the other hand, if DVT was suspected from the symptoms, venous ultrasonography of the lower limb was performed before contrast‐enhanced CT. Immediately after DVT was detected by venous ultrasonography of the lower limbs, contrast‐enhanced CT confirmed the presence of PE, and echocardiography confirmed the presence or absence of right heart failure. Acute DVT refers to venous thrombosis for which symptoms have been present for two weeks or for which imaging studies indicate that venous thrombosis occurred within the last 2 weeks.^11^ Nevertheless, the definition of acute PE is not described in Japanese and foreign guidelines and literature. Normally, an acute PE is due to lower limb DVT.^1^, ^2^ Based on the above definitions, in this study, the definition of acute DVT and PE refers to venous thrombosis for which symptoms have been present for two weeks or less or for which imaging studies indicate that acute DVT occurred within the last two weeks.^4^, ^5^ Conversely, there were patients who had symptoms but did not go to the hospital or were suspected of other diseases and had not been tested for more than 2 weeks. There were also excluded cases where imaging did not show that acute DVT had occurred within the last two weeks. Patients diagnosed with primary acute PE and DVT were included in the study, while those with DVT only were excluded. In addition, we excluded patients with a history of PE/DVT, cases demonstrating more than 2 weeks after symptom onset, unknown onset dates, interstitial pneumonia, or emphysema, as these factors may affect the distribution of PE and DVT, and right ventricular (RV) pressure.

### 2.2 Thrombus detection methods

Patients were scanned from the chest downwards using a Discovery 64 row multislice CT system (GE Healthcare, Boston, MA) for CTPA and CT venography. The pulmonary artery (from the apex to the inferior margin of the diaphragm) was scanned starting 25 s after the start of contrast agent injection (standard injection rate, 3 mL/s; concentration, 370 mg iodine/mL; injection volume, 100 mL), and the leg veins were scanned (from the inferior vena cava [IVC] to the ankle) 3.5 minutes later (Table 1).^12^ The amount of contrast agent administered was set at a total of 600 mg of iodine per kg of body weight. The amount was reduced at the discretion of the attending physician depending on the patient's general condition, such as the presence of right heart overload. To ensure adequate visualization of the lower leg veins (particularly the soleal vein), an object, such as a pillow, was placed under the heels during scanning to avoid compression of this area. In patients with severe PE, there was a possibility that the pulmonary circulation may be delayed, and the veins may not be contrast‐enhanced 3.5 minutes after the injection of the contrast agent. In such patients, additional scanning was performed if the initial enhancement was deemed to be poor.

**TABLE 1:** Main scanning protocols for CT pulmonary angiography and indirect CT venography

	CT pulmonary angiography	Indirect CT venography
Scanning range	Pulmonary artery: Apex‐diaphragm	Leg veins: Inferior vena cava‐ankle
Tube voltage (kV)	120 kV	
Current (mAs)	CT‐AEC used	
Scan slice thickness	0.625 mm	
Reconstruction slice thickness	0.625 mm	1.25 mm
Image display	Cross‐sectional images, MPR (coronal and oblique sagittal sections)	Cross‐sectional images, MPR (coronal section), CPR, magnified reconstruction to the ankle from the knee

Abbreviations: CPR, curved multi‐planar reconstruction; CT, computed tomography; CT‐AEC, computed tomography auto‐exposure control; MPR, multi‐planar reconstruction.

Venous ultrasonography of the lower limbs was performed using a Toshiba Aplio XV or Xario XG system (Toshiba Medical Systems, Tokyo, Japan) fitted with a 4.0‐11‐MHz linear probe. In accordance with the Japan Society of Ultrasonics in Medicine guidelines,^4^ the deep veins were compressed at 90º (short‐axis view) to their long axes in a continuous series, from the common femoral vein to the lower leg, and lack of venous compressibility and thrombotic echoes were identified in B mode. To improve diagnostic accuracy, examinations distal to the popliteal vein were, in principle, performed by the compression method with the patient in a seated position so as to engorge the veins fully.

### 2.3 Criteria for determining the presence or absence of thrombi

Thrombi were identified on CTPA and CT venography based on the presence of a contrast defect by a board‐certified diagnostic radiologist with > 10 years of experience.

We performed venous ultrasonography and diagnosis of the lower extremities according to widely used guidelines of the Japan Society of Ultrasonics in Medicine.^4^ At the time of the examination, both the compression method and the color Doppler method were used. As criteria for diagnosis of venous thrombosis, direct ultrasound signs of venous thrombosis were thrombotic echoes in veins, and lack of venous compressibility. Indirect signs are blood flow defects in the vein. The finding of a direct sign allowed a definite diagnosis of venous thrombosis. Individuals having only indirect signs were rated as being suspected of having venous thrombosis. And the results were interpreted by a diagnostic radiographer with > 4 years of experience in vascular ultrasonography and a board‐certified diagnostic radiologist with > 10 years of experience.

### 2.4 Echocardiography devices

Echocardiography was performed using a GE LOGIQ E9 XDclear 2.0 system (GE Healthcare, Milwaukee, WI, USA) fitted with a 1.5‐4.6‐MHz sector probe, a GE Vivid7 system (GE Healthcare) fitted with a 1.5‐4.0‐MHz sector probe, a Philips IE33 system (Philips Medical, Best, Netherlands) fitted with a 1‐5‐MHz sector probe, or a Philips SONOS 7500 system (Philips Medical) fitted with a 1‐3‐MHz sector probe.

### 2.5 Sites assessed for the presence of thrombi

We assessed the pulmonary artery using CTPA; the IVC, common iliac vein (CIV), and external iliac vein using CT venography; and the common femoral, (superficial) femoral, popliteal, posterior tibial, peroneal, and soleal veins using venous ultrasonography of the lower limbs for the presence of thrombi. Either of the above‐mentioned veins were defined as the most proximal site of DVT on the basis of criteria elaborated in the following section.

### 2.6 Definition of the most proximal site and laterality of DVT

If a thrombus was present in the IVC, the most proximal site of DVT was defined as follows: (a) if DVT was unilateral and thrombi were found in the IVC and CIV, the most proximal site was the IVC; (b) if DVT was bilateral and a thrombus was found in the IVC as well as CIV of one leg, two most proximal sites were defined, one in the IVC and another in the other leg; (c) if thrombi were present in the IVC and bilateral CIVs, the most proximal site was the IVC; and (d) if there was a localized thrombus in the IVC, the most proximal site was the IVC.

If a thrombus was present in a vein distal to the CIV, one most proximal site was defined if the thrombus was present in only one leg, whereas two were used if thrombi were present in both legs. As for laterality, DVT was defined as unilateral if the thrombus was present in the veins of one side, and as bilateral if thrombi were present in both legs distal to the CIV, whether or not a thrombus was present in the IVC.

### 2.7 Severity grading system for acute PE

The severity of acute PE was graded in accordance with the guidelines for the diagnosis, treatment, and prevention of PE and DVT (Table 2).^5^ "Cardiac arrest" was defined as cardiac arrest or circulatory ischemia in either case associated with signs of right heart failure on echocardiography; “massive” as hemodynamic instability with signs of right heart failure on echocardiography; “submassive” as hemodynamic stability with signs of right heart failure on echocardiography; and “nonmassive” as hemodynamic stability with no signs of right heart failure on echocardiography. Therefore, right heart failure is included in the diagnostic criteria of submassive or worse PE. Right heart failure was evaluated based on the presence of right ventricular dilatation (an increased end‐diastolic RV:LV (left ventricular) diameter ratio ≥ 1.0), hypokinesia of the free RV wall, and pulmonary hypertension (tricuspid systolic pressure gradient ≥ 40 mmHg) on echocardiography.^5^, ^13^, ^14^ The tricuspid systolic pressure gradient was calculated from the tricuspid regurgitation velocity using the continuous wave Doppler method. The results were interpreted by a clinical laboratory technician with > 10 years of experience in echocardiography and a board‐certified cardiologist with > 12 years of experience.

**TABLE 2:** Classification of clinical severity of acute pulmonary embolism^5^

	Hemodynamics	Right heart overload observed on echocardiography
Cardiac arrest collapse	Cardiac arrest or circulatory collapse	Present
Massive	Unstable Shock or hypotension (defined as a systolic blood pressure of < 90 mmHg last ≧15 min or a decrease in blood pressure by ≧40 mmHg, regardless of the presence/absence of new onset of arrhythmia, dehydration, or sepsis)	Present
Submassive	Stable (absence of the above findings)	Present
Nonmassive	Stable (absence of the above findings)	Absent

### 2.8 Parameters investigated

The associations between right heart failure and age, gender (male/female), PE distribution (one or both lungs), the most proximal site of DVT in the soleal vein (yes or no), DVT distribution (one or both legs), the most proximal site of DVT in the IVC or CIV (yes or no), and malignancy (present or absent) were investigated.

### 2.9 Statistical analysis

A logistic model was used for statistical analysis, and univariate and multivariate analyses were performed. The independent variables included in the multivariate analysis were chosen based on previous study findings and clinical judgment.^3^, ^9^, ^15^, ^16^ A univariate analysis of these independent variables was performed before the multivariate analysis. *P* values <.05 were considered to indicate statistical significance. All analyses were performed using EZR software (Jichi Medical University Saitama Medical Center, Saitama, Japan).

## 3 RESULTS

### 3.1 Patients

**FIGURE 1:**
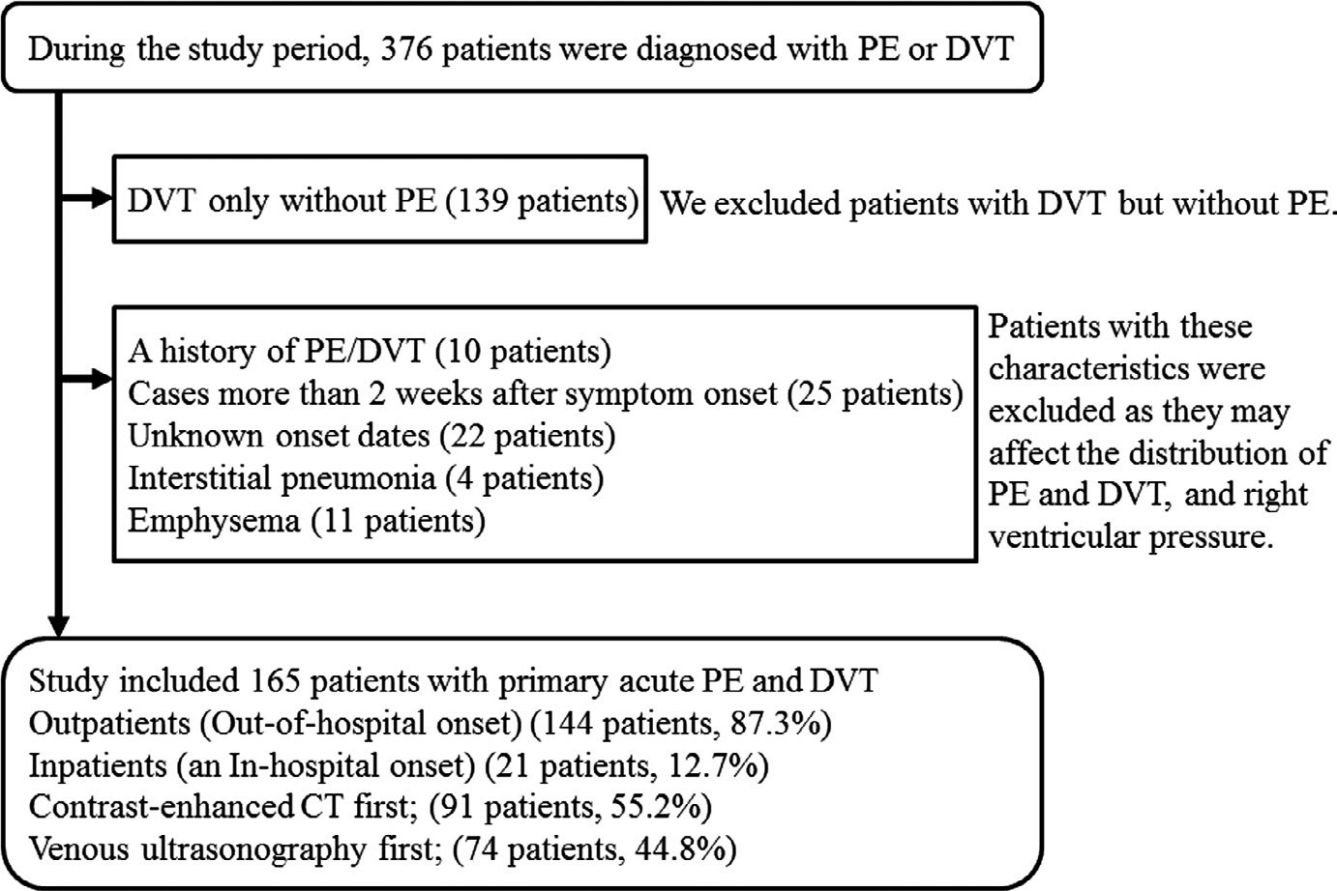
Participants and exclusion criteria

During the study period, 376 patients were diagnosed with PE or DVT. Patients with DVT only (139), a history of PE/DVT (10), cases more than 2 weeks after symptom onset (25), unknown onset dates (22), interstitial pneumonia (4), and emphysema (11) were excluded based on the aforementioned criteria. The study finally included 165 patients with primary acute PE and DVT (Figure 1), of which 77 were male (154 legs) and 88 were females (176 legs). The mean age was 65.1 ± 13.7 years. The outpatients (out‐of‐hospital onset) were 144 patients (87.3%). Patients hospitalized in our hospital or another hospital (an in‐hospital onset) were 21 patients (12.7%).

If PE was suspected from the symptoms, contrast‐enhanced CT was performed before venous ultrasonography of the lower limbs. The order of this examination was 91 (55.2%) of the 165 patients studied. Immediately after PE was detected by contrast‐enhanced CT, venous ultrasonography of the lower limbs confirmed the presence of DVT. On the other hand, if DVT was suspected from the symptoms, venous ultrasonography of the lower limb was performed before contrast‐enhanced CT. The order of this examination was 74 (44.8%) of the 165 patients studied. Immediately after DVT was detected by venous ultrasonography of the lower limbs, contrast‐enhanced CT confirmed the presence of PE, and echocardiography confirmed the presence or absence of right heart failure.

Based on clinical severity, 3 cases (1.8%) were graded as circulatory collapse, 2 (1.2%) as massive, 48 (29.1%) as submassive, and 112 (67.9%) as nonmassive acute PE.^5^ Right heart failure was present in 53 patients (32.1%). Table 3 shows the frequency of PE and DVT risk factors in the submassive or worse (53 patients) and nonmassive (112 patients) groups. Among the risk factors studied, acute PE classified as submassive or worse (acute PE with right heart failure) was found to be present in a significantly greater proportion of females (69.8% versus 45.5%, *P* = .004). In contrast, it was present in a significantly lower proportion of patients with malignancy (5.7% versus 19.6%, *P* = .035) (Table 3).

**TABLE 3:** The frequency of PE and DVT risk factors in the submassive or worse (53 patients) and nonmassive (112 patients) groups

	Submassive or worse (n = 53)	Nonmassive (n = 112)	*P*‐value
Age, mean ± SD	67.6 ± 12.3	64.0 ± 14.2	.095
Female gender, n(%)	37 (69.8%)	51 (45.5%)	.004
Bed rest > 3 d, n (%)	1 (1.9%)	2 (1.8%)	.96
Obesity (BMI ≧ 25.0 kg/m^2^), n(%)	21 (39.6%)	48 (42.9%)	.69
Pregnancy/childbirth, n(%)	1 (1.9%)	0 (0.0%)	N/A
Cerebrovascular disease, n(%)	0 (0.0%)	6 (5.4%)	N/A
Varicose veins in the lower limbs, n(%)	2 (3.8%)	10 (8.9%)	.39
Recent surgeries (within 1 mo), n(%)	2 (3.8%)	6 (5.4%)	.96
Recent trauma, fractures (within 1 mo), n(%)	1 (1.9%)	7 (6.3%)	.41
Central venous catheterization (femoral site), catheter test/intervention, n(%)	0 (0.0%)	1 (0.9%)	N/A
Malignant tumors, n(%)	3 (5.7%)	22 (19.6%)	.035
Benign abdominal tumors, n(%)	1 (1.9%)	2 (1.8%)	.56
Suspected congenital anticoagulant deficiency, n(%)	9 (22.5%)	9 (15.5%)	.54
Drugs (eg, oral contraceptives, estrogens), n(%)	7 (13.2%)	8 (7.1%）	.33
Outpatients (Out‐of‐hospital onset), n(%)	47 (88.7%)	97 (86.6%)	.71

Note

Student's *t* test was used for continuous variables, and a chi‐squared test was used for categorical variables.

Abbreviations: BMI, body mass index; BNP, blood natriuretic protein; N/A, not applicable; SD, standard deviation.

### 3.2 Factors associated with right heart failure in patients with primary acute PE and DVT (n = 165)

Univariate analysis revealed that the odds ratio (OR) for right heart failure was significantly lower in patients with IVC or CIV because the most proximal site of DVT (OR = 0.06, 95% confidence interval [CI] 0.01‐0.44, *P* = .005) and malignancy were present (OR = 0.25, 95% CI 0.07‐0.86, *P* = .028) (Table 4). Conversely, it was significantly higher in females (OR = 2.75, 95% CI 1.32‐5.94, *P* = .004) and in patients with venous thrombi in both legs (OR = 2.31, 95% CI 1.17‐4.53, *P* = .015) when the most proximal site of DVT was the soleal vein (isolated soleal vein thrombosis) (OR = 3.09, 95% CI 1.42‐6.71, *P* = .005).

**TABLE 4:** Factors associated with right heart failure in patients with primary acute PE and DVT (n = 165)

Factor	Univariate analysis			Multivariate analysis		
	OR	95% CI	*P* value	OR	95% CI	*P* value
Age	1.02	1.00‐1.05	.11	1.02	0.98‐1.05	.344
Female gender	2.75	1.32‐5.94	.004	2.51	1.05‐6.01	.039
Bilateral/unilateral PE	N/A	N/A	N/A	N/A	N/A	N/A
Most proximal site of DVT in the IVC or CIV (yes or no)	0.06	0.01‐0.44	.005	0.07	0.01‐0.62	.017
DVT in both legs/one leg	2.31	1.17‐4.53	.015	3.89	1.60‐9.48	.003
Most proximal site of DVT in the soleal vein (yes or no)	3.09	1.42‐6.71	.005	1.69	0.63‐4.55	.295
Malignancy (present or absent)	0.25	0.07‐0.86	.028	0.26	0.06‐1.23	.090

Abbreviations: CI, confidence interval; CIV, common iliac vein; DVT, deep vein thrombosis; IVC, inferior vena cava; N/A, not applicable; OR, odds ratio; PE, pulmonary thrombosis.

Multivariate analysis revealed that the odds ratio (OR) for right heart failure was significantly lower in patients with the most proximal site of DVT in the IVC/CIV (OR = 0.07, 95% CI 0.01‐0.62, *P* = .017) while it was significantly higher in females (OR = 2.51, 95% CI 1.05‐6.01, *P* = .039) and in patients who exhibited the presence of bilateral venous thrombosis (OR = 3.89, 95% CI 1.60‐9.48, *P* = .003).

### 3.3 DVT in the IVC or CIV

Since we found that the IVC or CIV as the most proximal site of DVT decreased the risk of right heart failure, we investigated thrombus location in patients without right heart failure. Of the 12 patients with the most proximal site of DVT in the IVC, the thrombus was continuous from the IVC to the soleal vein in 10 (83.3%) and continuous from the IVC to the peroneal vein in all 12 (100%). In the 16 patients in whom the most proximal site of DVT was in the CIV, the thrombus was found to be continuous from the CIV to the soleal vein in 10 (62.5%). The thrombus was found to be continuous from the CIV to the femoral vein in 5 (31.3%) patients and from the CIV to the common femoral vein in 1 (6.3%).

## 4 DISCUSSION

This was a single‐center retrospective study of factors associated with right heart failure in patients with primary acute PE and DVT.

A significant factor involved in PE without right heart failure was the most proximal site of DVT in the IVC/CIV, and significant risk factors associated with PE with right heart failure were shown to be in females and in patients with bilateral venous thrombosis.

Ohgi et al retrospectively examined PE after embolization and the most proximal site of DVT remaining in the lower limbs, and found that in patients with no right heart overload, the most common proximal sites of DVT were the CIV and external iliac vein.^15^ However, because they included chronic PE cases, and IVC and the lower thigh veins were not investigated in detail, the relationship between the risk of right heart overload in primary acute PE and the most proximal site of DVT remaining in the lower limbs is not fully understood.

Because we found that a significant factor involved in PE without right heart failure to be the most proximal site of DVT in the IVC or CIV, we assessed the thrombus location in the patients without right heart failure. Of the 12 patients with a most proximal site of DVT in the IVC, the thrombus was continuous from the IVC to the soleal vein in 10 (83.3%) and from the IVC to the peroneal vein in all 12 (100%). It is likely that the thrombus originated in the soleal vein and extended proximally to the IVC in all 12 patients because the IVC itself does not contain any points of origin, unlike the CIV, which may be affected by iliac compression syndrome. We believe that the thrombus embolized during proximal migration to the IVC, leading to nonmassive PE that does not cause right heart failure. In the 16 patients in whom the most proximal site of DVT was in the CIV, the thrombus was found to be continuous from the CIV to soleal vein in 10 (62.5%), from the CIV to the femoral vein in 5 (31.3%), and from the CIV to the common femoral vein in 1 (6.3%). The thrombus may have occurred in the soleal vein and migrated to the proximal veins, reaching the CIV. Alternatively, it may have originated from the CIV and migrated to the distal veins. Similar to the patients with the most proximal site in the IVC, the thrombus is presumed to have embolized during migration, leading to nonmassive PE with no right heart failure. When the thrombus is not fixed to the vein wall, the thrombus within the IVC and CIV may detach due to a change in body position (change in blood flow).^3^, ^16^ However, acute thrombi are anchored by inflammatory changes in the venous wall after a few days; therefore, they are less likely to detach after this period.^17^ Furthermore, it is thought that it takes a longer time for a thrombus to migrate proximally from the soleal vein to the CIV and beyond, as compared with migration from the soleal vein to the popliteal vein. In addition, most cases of iliac venous thrombosis have been reported to become organized earlier on the proximal side and adhere to the venous wall at the same time as they migrate distally, which both decreases the likelihood of embolization and makes it harder for free‐floating thrombi to form.^16^, ^18^ Thus, our finding that a significant factor involved in PE without right heart failure was the most proximal site of DVT in the IVC/CIV might indirectly reflect the fact that these clots were attached to the venous wall. They were, therefore, less likely to detach, thereby reducing the possibility of obstructing the pulmonary circulation.

We also found that a significant risk factor associated with PE with right heart failure was the presence of bilateral venous thrombi. When venous thrombi are present in both legs rather than only one, the volume of the thrombus and the area which it occupies increases. In this case, there is a significant possibility that the area of the pulmonary artery that is embolized may be increased. If compensation is inadequate, right heart failure may occur. There were no deaths recorded in our study. Ro et al retrospectively examined 100 patients who died of PE, finding that 89% had bilateral lower extremity residual thrombi.^9^ The cases mentioned in the report all resulted in death, which is different from the PE severity of our study. However, this previous study indicated that the presence of bilateral thrombi was involved in right heart failure, and it could even lead to death.

All the 165 patients in this study were PE and DVT patients, of which 74 (44.8%) underwent venous ultrasonography of the lower limb before contrast‐enhanced CT. Immediately after DVT was detected by venous ultrasonography of the lower limbs, contrast‐enhanced CT confirmed the presence of PE, and echocardiography confirmed the presence or absence of right heart failure. In the situation where the presence of PE and right heart failure had not been confirmed by testing, if venous thrombus of both legs was present on venous ultrasonography of the lower limb, the possibility of right heart failure because of PE complications could be considered. It could be used as a criterion to decide whether to perform contrast‐enhanced CT and echocardiography. In addition, even in clinics where contrast‐enhanced CT or echocardiography could not be performed, the distribution of lower‐limb thrombus by venous ultrasonography of the lower limb might be used as a criterion for determining whether it should be immediately introduced to a specialized hospital. Therefore, it is meaningful to investigate factors associated with right heart failure in patients with primary acute PE and DVT after embolization, especially the residual thrombus in the lower limbs.

We recognize that there are some limitations to this study. First, this was a single‐center retrospective study with a limited sample size, which may contribute to bias. In addition, the effect of the amount of clots on the severity of PE could not be investigated since thrombi could not be quantified. This may limit the generalizability of the results. In order to confirm our findings, it is necessary to conduct larger multicenter collaborative studies. We clarified the relationship between the risk of right heart failure and the residual thrombus site in the lower extremities in primary acute PE. In the future, it is necessary to examine the relationship between the distribution of PE and DVT, identify predictors of the clinical course, and determine whether invasive examination and treatment can be avoided.

## 5 CONCLUSION

A significant factor involved in PE without right heart failure was the most proximal site of DVT in the IVC/CIV, and significant risk factors associated with PE with right heart failure were more prevalent in females and in patients who exhibited the presence of bilateral venous thrombosis.

## CONFLICT OF INTEREST

The authors have stated explicitly that there are no conflict of interest in connection with this article.
